# Circular saw misuse is related to upper limb injuries: a cross-sectional study

**DOI:** 10.6061/clinics/2019/e1076

**Published:** 2019-09-04

**Authors:** Rodrigo Guerra Sabongi, Jaime Piccaro Erazo, Vinicius Ynoe de Moraes, Carlos Henrique Fernandes, João Baptista Gomes dos Santos, Flávio Faloppa, João Carlos Belloti

**Affiliations:** Disciplina de Cirurgia da Mao e Membro Superior, Departamento Ortopedia e Traumatologia, Universidade Federal de Sao Paulo, Escola Paulista de Medicina, Sao Paulo, SP, BR

**Keywords:** Wounds and Injuries, Hand Injuries, Accident Prevention, Cross-Sectional Study

## Abstract

**OBJECTIVES:**

Machinery injuries account for a substantial share of traumatic upper limb injuries (TULIs) affecting young active individuals. This study is based on the hypothesis that there is an important relationship between the improper use of power saws and TULIs. The aim of the study is to assess the prevalence and epidemiology of TULIs caused by power saws and determine the risks related to power saw use.

**METHODS:**

A cross-sectional evaluation of medical records from a two-year period was performed. Patients sustaining TULIs related to power saws were analyzed. Data on the epidemiology, site of injury, mechanism of trauma, technical specifications of the tool, cutting material, personal protective equipment, time lost and return to work were obtained.

**RESULTS:**

A database search retrieved 193 TULI records, of which 104 were related to power saws. The majority of patients were male (102/104; 98.1%), right-handed (97/104; 93.3%), and manual workers (46/104; 44.2%), with an average age of 46.8 years. The thumb was the most frequently injured site (32/93; 34.4%). Most of the injuries were caused by manual saws (85/104; 81.7%), and masonry saws accounted for 68.2% (58/85) of the cases. Masonry saws improperly used for woodwork resulted in 86.2% (50/58) of the injuries. TULI caused by masonry saws was 5 times higher in manual workers than in other patients. In addition, masonry saws had a risk of kickback 15 times higher than that of other saws, and the risk of injury increased by 5.25 times when the saws were used improperly for wood cutting.

**CONCLUSIONS:**

The profile of TULIs related to power saws was demonstrated and was mainly associated with manual saws operated by manual workers that inappropriately used masonry saws for woodworking.

## INTRODUCTION

The upper limbs are the locations most affected by trauma 1. A high prevalence of upper limb injuries is present in all industrialized countries. Industrial machine operation predisposes workers to severe and incapacitating injuries that have become ordinary in the modern world 2. Frequently, these injuries are related to the inappropriate use of powerful tools, such as the removal of protection devices, nonergonomic operation and cutting of materials not suited for the machine. Despite this knowledge, no study has sought to explore this correlation in Brazil.

Machinery injuries account for a substantial share of traumatic upper limb injuries (TULIs). Young active patients and manual workers are particularly predisposed to hand lesions 3. Studies evaluating work-related TULIs revealed that 16.5% of injuries were caused by circular saws 4, and 56.8% were caused by machinery in general 5. Other studies found that machinery and power saws accounted for 14% to 34% of traumatic injuries 3,6. Power saws are frequently associated with severe hand injuries, mainly through blade contact, resulting in significant tissue destruction. These injuries result in amputation in 57% of the affected trauma patients, demonstrating the severity of injuries caused by high-energy contact.

The relevance of the problem is represented by not only its elevated prevalence but also its socioeconomic impact. The primary costs related to patient care and injury treatment are always coupled with enormous secondary burdens, which include lost wages, job readjustments and permanent disability. The period of rehabilitation must be considered in the analysis of the economic impact of injuries since this may be associated with the most significant loss 3,7,8. A study estimated an average of 64 days off from work after circular saw injuries, with a mean cost of over 30 thousand dollars per injury 9. In addition, the sequelae of these injuries generate long-lasting functional impairment that affects the patient's quality of life 8,10.

Prevention strategies can be elaborated after a thorough analysis of the lesions, the mechanisms of trauma and the demographic characteristics, with emphases on the direct and indirect aspects related to the operator. There is scarce information in the literature about TULIs and the risks related to the inappropriate use of circular saws. Although safety regulations have had a long-term impact 11, the prevalence of these injuries remains high; an analysis of their characteristics and the development of new preventive measures warrant attention.

This study is based on the hypothesis that there is an important relationship between the improper use of power saws and TULIs. The aim of the study is to verify the prevalence and epidemiology of TULIs caused by circular saws and to determine the risks related to circular saw use and misuse.

## MATERIALS AND METHODS

This study was designed as a cross-sectional analysis, and the reporting of results followed the Strengthening the Reporting of Observational Studies in Epidemiology (STROBE) statement guidelines 12. The study was performed in a single center (Hand and Upper Limb Surgery section of the Escola Paulista de Medicina – Federal University of São Paulo) and involved the evaluation of outpatients during a period of two years (May 2014 to May 2016). The study was self-funded by the authors, and all patients were treated by multiple surgeons from the university faculty. Records with information consistent with TULI caused by machinery were selected from the electronic medical record system. The institutional electronic record system contains mandatory International Classification of Diseases (ICD-10) codes for all treated patients as well as detailed information about trauma and injury agents. Therefore, medical records could be indexed by ICD-10 codes, and the codes related to penetrating injuries and their complications were selected. The database was analyzed separately by two authors.

### Inclusion criteria

Patients with complete medical record information and ICD-10 codes for wounds, tendon injuries, neurovascular injuries, amputations and sequelae from trauma caused by power saws were included.

### Exclusion criteria

Patients who could not be contacted and who did not consent to the study were excluded. In addition, those in which the etiology of injury was related to industrial machines (presses, gears, dropping of industrial objects, etc.) with avulsion or crushing mechanisms were excluded.

### Data collection

Eligible patients were contacted and invited to answer a questionnaire after informed consent was obtained. All patients were contacted by the main author. Four attempts were made on different days and times before the record was excluded from the study. Epidemiological data (sex, age, dominant hand, profession), the site of injury and the mechanism of trauma were obtained.

Professions were categorized as follows: (1) manual worker; (2) artisan; (3) office or service worker; (4) merchant; (5) technician; (6) student; (7) farmer and (8) retired or unemployed. The trauma mechanisms were categorized according to the event that generated the contact with the blade: (1) kickback; (2) machine and/or material slippage; (3) disk breakage; (4) contact during a cutting procedure; (5) attention distraction during operation; (6) operator's clothing caught in the saw; (7) machine cleaning; (8) contact after turning off the tool; (9) unbalance or fall by the operator.

Stationary saws in which the blade is positioned in a table or stand, such as circular table saws, band saws, surface planers and table jointers, were considered power tools. Manual saws, such as hand wood circular saws, masonry saws, grinders, hole saws, portable planers and brush cutters, were defined as power tools, as the operator handles the cutting disk.

In addition, information was collected regarding the technical specifications of the instrument, cutting material, personal protective equipment (PPE), time to return to work and time lost from work (in months). The return to work was defined as the time when the patient was able to perform the same activities as those performed prior to the accident. After the use of the power saw was described, the patient was questioned about their knowledge of the appropriate application of the machine for the intended activity to verify the inadequate use of power saws.

### Statistical analyses

The statistical analysis of the baseline data was performed was performed to obtain mean, minimum and maximum values; standard deviations (SDs); and frequencies. For the proportions of interest, 95% confidence intervals (CIs) were calculated. The odds ratios (ORs) for the different modalities of saws and the contributing factors was calculated with 2x2 contingency tables and subsequent calculations for each of the situations of interest. For these analyses, the nonrisk group was considered a control for the exposed cases. The differences between the distinct groups were compared using the chi-square test. Student's t-test was used to calculate the difference between the means in the comparison groups. An alpha was considered significant at less than 5%.

### Ethics

Patients enrolled in this study gave oral consent to participate. Approval from the Federal University of São Paulo Ethics Committee was recorded under the registry number 61257216100005505.

## RESULTS

**Figure 1:**
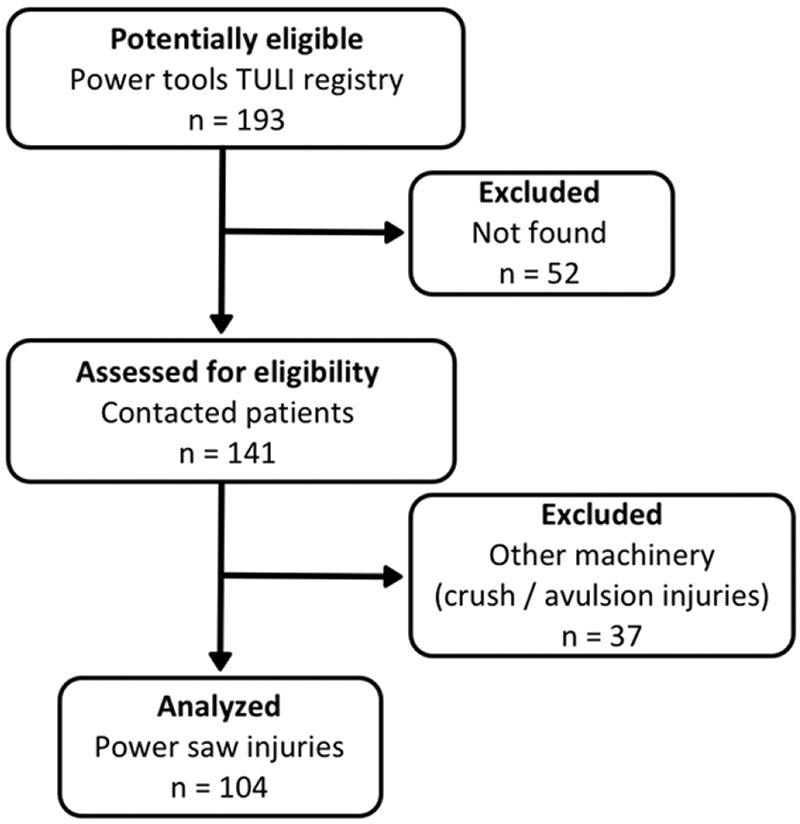
Study flowchart.

Patient selection resulted in 193 records of TULI caused by machinery; 141 contacts were available to complete the preestablished questionnaire. No patient declined to enroll in the study. Thirty-seven patients were excluded due to avulsion or crushing injuries (due to gears, cogwheels, hydraulic presses, among other industrial machines). Therefore, the total number of patients with TULIs related to power saws was 104 (Figure 1). The majority of patients were male (102/104; 98.1%), right-handed (97/104; 93.3%), and manual workers (46/104; 44.2%), with a mean age of 46.8 years (ranging from 15-73 years, median=48; IQR=19). The labor categories are depicted in Table 1.

**Figure 2:**
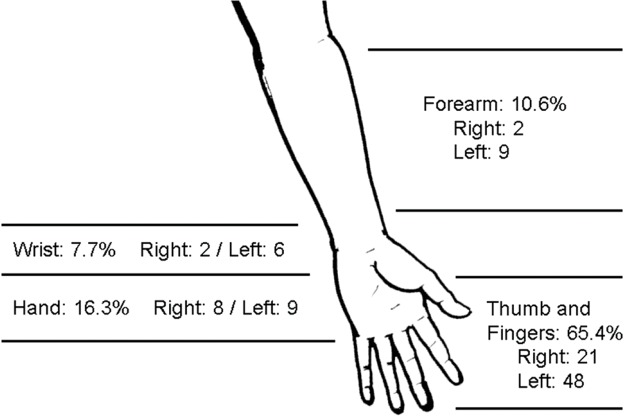
Location of injuries in 104 patients.

Most of the lesions were located on the fingers (Figure 2) and most commonly involved a single finger. The thumb was the most frequently injured site (32/93; 34.4%), usually on the left side. The detailed characteristics of the 93 injured fingers are shown in Table 2.

Manual saws accounted for the majority of the injuries, accounting for 81.7% (85/104) of the cases, while 18.3% (19/104) were related to stationary saws. Among the latter, the most common causes were table circular saws in 78.9% (15/19) of the cases, followed by band saws (2/19; 10.5%), surface planers (1/19; 5.3%) and table jointers (1/19; 5.3%).

**Figure 3:**
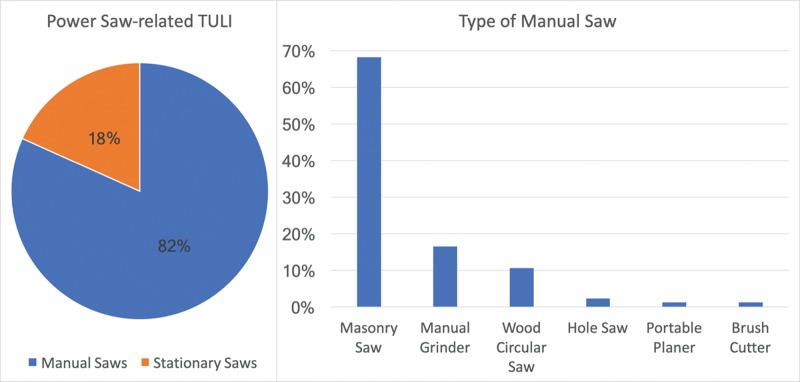
Saw-related TULIs and the type of manual saw.

Among the manual saws, most of the injuries resulted from accidents with masonry saws (58/85; 68.2%), followed by grinders (14/85; 16.5%), wood circular saws (9/85; 10.6%), hole saws (2/85; 2.3%), portable planers (1/85; 1.2%) and brush cutters (1/85; 1.2%). The prevalence of saw-related TULIs is summarized in Figure 3.

Of the 58 cases related to masonry saws, 50 resulted from cutting wood (86.2%; CI=[77.3%, 95.1%]); all of the patients improperly used wood-cutting wheels adapted to the tool. When asked about their knowledge about proper application of masonry saws, 34/58 (58.6%) knew that its use for wood cutting was inappropriate.

The most common mechanism that produced the injuries was kickback (49/104; 47.1%), a phenomenon that occurs when the blade lifts the cutting material through a sudden lock with displacement toward the operator 13. The other mechanisms are summarized in Table 3.

The use of PPE at the time of the accident was reported by 69/104 (66.3%) patients. The risk (OR) of TULI was not related to the use of PPE (OR=0.77, 95% CI=0.3-1.7, *p*=0.52) when comparing manual and stationary saws. Likewise, for comparison of masonry saw with other saws, the use of PPE was not related to an increased risk (OR=0.84, 95% CI=0.3-2.4, *p*=0.74).

The majority of patients returned to the same work performed before the accident (64/104; 61.5%), with a mean of 6.8 months (median=6; IQR=9) of remission. When stratified by the type of saw, masonry saw accounted for an average time lost from work of 12.5 months (median=6; IQR=15), while the other saws accounted for an average of 10.9 months (median=5; IQR=8). There was no significant difference in the time away from work associated with masonry saws and other saws (Student's t=0.44; *p*=0.66).

The risk (OR) of TULI caused by masonry saws was 5 times higher in manual workers than in other patients (OR=5.0, 95% CI=2.1-11.7, *p*=0.0002). In addition, masonry saws presented a risk of kickback 15 times higher than that of other saws (OR=15.0, 95% CI=5.6-40.3, *p*<0.0001), and the risk of injury increased by 5.25 times when the saws were used improperly for wood cutting (OR=5.25, 95% CI=2.0-13.5, *p*=0.0006). The results of the ORs are consolidated in Table 4.

## DISCUSSION

The high frequency of saw injuries and the details of their characteristics were analyzed in this study. The number of participants was consistent with the literature 8,9,14, with a representative sample from a quaternary hospital. Unlike other studies 1,2,15, we found a high frequency of injuries related to manual saws (81.73%), which may reflect the profile of national construction and domestic activities. In our country, the use of stationary saws has decreased due to the use of new building materials instead of wood resources. In addition, overall construction is not primarily based on wood, and many materials are received with premade factory cuts. On the other hand, hand saws have gained popularity for their use in minor cuts and adjustments onsite, as well as their widespread availability for nonoccupational projects, such as recreational woodworking. Due to their portability, versatility, practicality, lightness and ease in producing small cuts, manual saws are improperly used to cut a range of materials that are often not suitable for the technical characteristics of the tool 16.

Work-related injury reports are essential for developing and planning effective preventive strategies 17. The data generated can be used to elaborate studies that reflect the real socioeconomic impact of accidents and outline valid prevention strategies. However, underreporting is an inherent problem for several reasons, such as doubts about compensation eligibility; concerns about reputation, career, and employer retaliation; and even underestimation of the severity of the injury 18-21. Nonoccupational injuries, where notification becomes more neglected, deserve attention due to their significantly increasing prevalence and the involvement of different ages and professions 13. A potential limitation of the study is the assessment of patients treated in a hand surgery unit, which may not be representative of the entire country's injury patterns. There might be a concern regarding memory bias; however, it is less probable that patients may forget such a dramatic limb-threatening event.

We observed sex and age characteristics similar to those of other studies, with injuries predominant in economically stable and active men 1,2,4,13,15. Manual saws were responsible for the majority (81.73%) of injuries, emphasizing their high usage rate. Studies from around the world have demonstrated that power saws are related to severe trauma in the upper limbs, and the mechanisms by which these accidents occur should be better elucidated 8-10,14,21.

Blade contact is the most common scenario, causing serious injuries, such as amputations, in both the lumber industries and the home environment 13,21. A well-described phenomenon is kickback, in which the machine or the cutting material experiences a sudden increase in resistance with unexpected and uncontrolled recoil toward the operator. Disks may jump or break under these conditions. Kickback generally results from the inappropriate use, such as improper operating procedures or conditions, of the power tool. Kickback was the most common trauma mechanism in our study, occurring in 47.1% of cases, which is consistent with the literature 8,13,14,22,23. Table saws have safety regulations requiring safety devices to avoid these accidents; however, they are frequently removed by operators for better visualization 11,24.

Manual and portable saws are prone to inappropriate use and risky operation, such as cutting without support, cutting at an inappropriate height, not balancing material, and especially cutting materials that are not intended for the tool. The masonry saw is a very popular professional and domestic hand-held device due to its wide availability, low cost, ease of operation and portability. According to regulatory standards, this power tool was specifically designed for cutting stones, bricks, concrete, masonry, vitreous materials and ceramic coatings 25. Its powerful motor is able to achieve high rotations with less torque than larger diameter circular saws; thus, it is not designed for woodworking. Its recommended use in conjunction with diamond cut-off wheels is usually modified with wood blades that can be adapted to the saw despite their technical incompatibility.

This saw was the most frequent agent in our study, causing 55.8% of all the injuries. It was used for cutting wood in 86.2% (CI=[77.3%, 95.1%]) of cases, and all injuries were caused by wood blades improperly adapted to the tool. Observing the lower limit of the CI (77.3%), it is notable that the inadequate use of the marble saw may represent the majority of injuries caused by this device in the population of interest. In addition, we found that the frequency of lesions due to woodworking with masonry saws was higher than that of any other saw used for cutting wood (86% *vs* 54%, X^2^: 12,949, *p*=0.0003), resulting in a 5.25 times higher risk of TULI.

Prevention is the key to reducing saw-related injuries since 85% to 96% of injuries could be prevented 9; safety regulations have demonstrated success in reducing the incidence of saw accidents, especially in the young population 11. Direct prevention strategies involve the operator's awareness about the proper use of the tool. In the work environment, this can be achieved through regular training, the use of PPE, the application of ergonomic principles and an improvement in work organization 22. However, for nonoccupational injuries, these measures are ineffective, and awareness is limited to the product's instructions for use, which are often ignored by the consumer. We found a high frequency of patients who were aware of the inappropriate use of the marble saw (59.65%), reflecting the inefficacy of a direct prevention strategy for this piece of equipment. Indirect approaches help prevent accidents independent of the operator's understanding and tend to be more effective in both occupational and home environments. Among them, we highlight the safety devices that aim to avoid blade contact by kickback. After the establishment of regulations that require their use, there was substantial reduction in the incidence of injuries 11,23,24.

Limitations of the study include its cross-sectional design with a small sample size from a single hand surgery center. It was not possible to select a control group of uninjured patients to typically calculate risk, and the calculated odds may still be underestimated. These results may not reflect reality with extreme precision. However, this study could serve as a pilot study for prospective trials that monitor power tools injuries focusing on the frequency of injuries related to specific types of saws.

The typical scenario of TULI caused by saws in our study was a male manual worker who improperly utilized a masonry saw for woodworking. In this regard, the authors propose an indirect prevention strategy that could be very effective: modify the disk socket to make wood blades incompatible with masonry saws. User manuals of the most popular masonry saws on the market directly specify that their use for wood cutting is inappropriate. The manuals also alert the consumers to use only diamond cut-off wheels for the power tool and that just because other accessories can be attached to the saws, their safe operation is not assured. Although regulatory standards define procedures for noise management, ergonomic aspects, and debris generation among others 26, there are no stipulations regarding the proper use of masonry saws and the manufacturing of compatible disks. A formal regulation for different saw and blade sockets would effectively prevent the use of saws fitted with improper disks, forcing operators to use the appropriate tool for the procedure. Secondary measures include stating the indications of use on the equipment box, with a clear description of the risks of improper usage; training sellers and power tool retailers; and infractions to employers that authorize equipment misuse. For policy makers, elementary actions such as these could have reduced more than half of the TULIs reported in our study, drastically reducing the social and economic impact of these injuries.

## CONCLUSIONS

The profile of TULIs related to power tools was demonstrated in this study. Injuries were caused mainly by hand-held saws and saws operated by manual workers. The improper use of masonry saws for wood cutting was the main cause of injury. In this study, the use of PPE did not seem to impact the risk of TULI.

## AUTHOR CONTRIBUTIONS

Sabongi RG and Erazo JP acquired the data and analyzed and interpreted the power saw injuries database. de Moraes VY, Fernandes CH, dos Santos JBG, Faloppa F and Belloti JC made substantial contributions to the study conception and design, and the analysis and interpretation of the data. Sabongi RG, de Moraes VY and Belloti JC were involved in writing the manuscript and revising it for publication. All authors have read and approved the final version of the manuscript.

## Figures and Tables

**Table 1:** Labor activities of the patients.

Profession	N	%
Manual worker	46	44.2
Artisan	14	13.5
Office / service worker	13	12.5
Unemployed / retired	10	9.6
Merchant	9	8.65
Technician	9	8.65
Student	2	1.9
Farmer	1	1.0

**Table 2:** Distribution of the 93 analyzed fingers.

Number of fingers	Thumb	Index	Middle	Ring	Minimum	Total
1	31	6	5	3	5	50
2	1	4	8	9	4	26
3	0	2	3	3	1	9
4	0	2				2
Total fingers	32	14	18	17	12	93
%	34.4	15.0	19.4	18.3	12.9	100

**Table 3:** Trauma mechanisms of the 104 power saw injuries.

Trauma mechanism	N	%
Kickback from the saw or cutting material	49	47.1
Saw / cutting material slippage	14	13.5
Disk breakage	10	9.6
Contact during the cutting procedure	10	9.6
Attention distraction during operation	8	7.7
Operator’s clothing caught in the saw	4	3.8
Machine cleaning	3	2.9
Contact after turning off the saw	3	2.9
Unbalance or fall by the operator	3	2.9

**Table 4:** ORs of TULIs caused by power saws.

Type of saw	OR	95% CI	*p*-value
Manual × stationary saw (control)	With × without PPE 0.77	0.3-1.7	0.52
Masonry saw × other saws (control)	With × without PPE 0.84	0.3-2.4	0.74
Masonry saw × other saws (control)	Manual worker × other professions 5.0	2.1-11.7	0.0002
Masonry saw × other saws (control)	Kickback × other mechanisms 15.0	5.6-40.3	<0.0001
Masonry saw × other saws (control)	Wood cutting × other materials 5.25	2.0-13.5	0.0006

## References

[1] Panagopoulou P, Antonopoulos CN, Dessypris N, Kanavidis P, Michelakos T, Petridou ET (2013). Epidemiological patterns and preventability of traumatic hand amputations among adults in Greece. Injury.

[2] Jin K, Lombardi DA, Courtney TK, Sorock GS, Li M, Pan R (2010). Patterns of work-related traumatic hand injury among hospitalised workers in the People’s Republic of China. Inj Prev.

[3] Trybus M, Lorkowski J, Brongel L, Hladki W (2006). Causes and consequences of hand injuries. Am J Surg.

[4] Pardini Junior AG, Tavares KE, Fonseca Neto JA (1990). Lesões da mão em acidentes de trabalho: análise de 1.000 casos. Rev Bras Ortop.

[5] Souza MA, Cabral LH, Sampaio RF, Mancini MC (2008). Acidentes de trabalho envolvendo mãos?: casos atendidos em um serviço de reabilitação. Fisioter Pesqui.

[6] Fonseca MC, Mazzer N, Barbieri CH, Elui VM (2006). Hand injuries: a retrospective study. Rev Bras Ortop.

[7] O’Sullivan ME, Colville J (1993). The economic impact of hand injuries. J Hand Surg Br.

[8] Frank M, Hecht J, Napp M, Lange J, Grossjohann R, Stengel D (2010). Mind your hand during the energy crunch: Functional Outcome of Circular Saw Hand Injuries. J Trauma Manag Outcomes.

[9] Hoxie SC, Capo JA, Dennison DG, Shin AY (2009). The economic impact of electric saw injuries to the hand. J Hand Surg Am.

[10] Kovacs L, Grob M, Zimmermann A, Eder M, Herschbach P, Henrich G (2011). Quality of life after severe hand injury. J Plast Reconstr Aesthetic Surg.

[11] Vosbikian MM, Harper CM, Byers A, Gutman A, Novack V, Iorio ML (2017). The Impact of Safety Regulations on the Incidence of Upper-Extremity Power Saw Injuries in the United States. J Hand Surg Am.

[12] Vandenbroucke JP, von Elm E, Altman DG, Gøtzsche PC, Mulrow CD, Pocock SJ (2007). Strengthening the Reporting of Observational Studies in Epidemiology (STROBE): explanation and elaboration. PLoS Med.

[13] Shields BJ, Wilkins JR 3rd, Smith GA (2011). Nonoccupational table saw-related injuries treated in US emergency departments, 1990-2007. J Trauma.

[14] Frank M, Lange J, Napp M, Hecht J, Ekkernkamp A, Hinz P (2010). Accidental circular saw hand injuries: trauma mechanisms, injury patterns, and accident insurance. Forensic Sci Int.

[15] Justis EJ, Moore SV, LaVelle DG (1987). Woodworking injuries: an epidemiologic survey of injuries sustained using woodworking machinery and hand tools. J Hand Surg Am.

[16] Barbosa AA (2014). Implementação de método de potência sonora em serra-mármore. Tese (doutorado). Universidade Estadual de Campinas.

[17] Division of Hazard and Injury Data Systems (2000). The National Electronic Injury Surveillance System A Tool for Researchers. US Consum Prod Saf Comm.

[18] Galizzi M, Miesmaa P, Punnett L, Slatin C (2010). Injured Workers’ Underreporting in the Health Care Industry: An Analysis Using Quantitative, Qualitative, and Observational Data. Ind Relat.

[19] Tonozzi TR, Marsh SM, Reichard AA, Bhandari R (2016). Reported work-related injuries and illnesses among Hispanic workers: Results from an emergency department surveillance system follow-back survey. Am J Ind Med.

[20] Tucker S, Diekrager D, Turner N, Kelloway EK (2014). Work-related injury underreporting among young workers: prevalence, gender differences, and explanations for underreporting. J Safety Res.

[21] de Souza V, Blank VL, Calvo MC (2002). [Scenarios of typical occupational injuries in lumber industry]. Rev Saude Publica.

[22] Holcroft CA, Punnett L (2009). Work environment risk factors for injuries in wood processing. J Safety Res.

[23] Beery L, Harris JR, Collins JW, Current RS, Amendola AA, Meyers AR (2014). Occupational injuries in Ohio wood product manufacturing: a descriptive analysis with emphasis on saw-related injuries and associated causes. Am J Ind Med.

[24] Chung KC, Shauver MJ (2013). Table saw injuries: epidemiology and a proposal for preventive measures. Plast Reconstr Surg.

[25] Australian/New Zealand StandardTM (2009). Hand-held motor-operated electric tools - Safety- Part 1: General requirements (IEC 60745-1 Ed 4, MOD).

[26] Norma Regulamentadora N° 12 - Segurança no Trabalho em Máquinas e Equipamentos (2015). http://trabalho.gov.br/seguranca-e-saude-no-trabalho/normatizacao/normas-regulamentadoras/norma-regulamentadora-n-12-seguranca-no-trabalho-em-maquinas-e-equipamentos.

